# Understanding and Improving Athlete Mental Health: A Social Identity Approach

**DOI:** 10.1007/s40279-024-01996-4

**Published:** 2024-02-26

**Authors:** Mark Stevens, Tegan Cruwys, Lisa Olive, Simon Rice

**Affiliations:** 1 School of Medicine and Psychology, The Australian National University, Canberra, ACT 2601 Australia; 2 School of Psychology, Deakin University, Geelong, VIC 3220 Australia; 3 IMPACT Institute for Mental and Physical Health and Clinical Translation, Deakin University, Geelong, VIC 3220 Australia; 4 Centre for Youth Mental Health, The University of Melbourne, Melbourne, VIC Australia; 5 Elite Sports and Mental Health, Orygen, 35 Poplar Road, Melbourne, VIC Australia

## Abstract

Understanding and positively influencing athlete mental health have become key goals for researchers and sporting stakeholders (e.g. coaches, support staff, clubs and governing bodies). In this article, we outline a novel perspective for tackling these challenges, drawing on an influential theory of group processes. This social identity approach can, we argue, help explain when and why the characteristics and demands of sport, which is typically a collective endeavour, pose a threat to athlete mental health and provide a guiding framework for efforts to protect and enhance athlete mental health. Here, we seek to illustrate the value of a social identity analysis of athlete mental health through three key points that speak to its analytical and practical value. Specifically, we propose: (1) that social identities can act as psychological resources that support athlete mental health, (2) that social identities are critical to athlete mental health during and after sporting transitions and (3) that leadership informed by a social identity approach can facilitate athlete mental health. With a view to maximising the value of our analysis both for those working with athletes and for researchers, we also identify practical steps that relevant stakeholders could take to support athlete mental health, and key avenues for future research to further test our propositions and advance understanding. Our analysis provides a new lens through which all those invested in understanding and supporting athlete mental health can approach these challenges, and a foundation for novel solutions.

## Key Points

**Table:** 

We outline a new perspective through which to view and tackle athlete mental health challenges, drawing on an influential theory of group processes: the social identity approach.
We illustrate the analytical and practical value of a social identity analysis of athlete mental health. In the process, we identify both practical steps that relevant stakeholders can take to support athlete mental health, and key avenues for future research to further test our propositions and advance understanding.
Our discussion focuses on how social identities can provide a key mental health supporting resource for athletes, the vital role social identities can play in supporting athlete mental health during sporting transitions, and the capacity for sporting leaders to foster athlete mental health through social identity leadership.

## Introduction

In recent times, athlete mental health has increasingly become part of the public consciousness. During the Tokyo Olympics, for example, it took centre stage when Simone Biles—one of the most high-profile athletes at the games—withdrew from competition citing mental health concerns and many other athletes revealed mental health struggles [1]. Athlete mental health has also become an increasing priority for researchers and policy makers. Indeed, there have been nine mental health position statements published by sport governing bodies since 2018 [2] and several recent attempts to synthesise and guide athlete mental health research (e.g. [3–5]). Researchers’ interest in athlete mental health has been driven by a growing awareness of the prevalence of mental ill-health among athletes, and of the sport-specific risk factors for mental ill-health that athletes face. Alongside the general stressors that athletes share with the general population (e.g. adverse life events), additional factors that pose a specific threat to athletes’—particularly elite athletes’—mental health include competition for selection, physical injuries and poor performance [6, 7]. A recent meta-analysis estimated that 34% of current elite athletes experience depression or anxiety, 26% experience sleep disturbance and 20% experience psychological distress [8]—prevalence rates that are at least comparable to those in the general population (see also [5, 9, 10]). Indeed, these statistics likely underestimate the problem. Stigma, low mental health literacy and busy schedules are among the many barriers to athletes seeking diagnosis and support for mental health concerns [11, 12]. In this article, we argue that efforts to understand and improve athlete mental health will be fruitfully enhanced by tackling these challenges through the lens of the social identity approach [13, 14]. This is not least because the social identity approach provides a comprehensive analysis of how people’s individual behaviours and psychology, including their mental health, are shaped by *group life*. The social identity framework can therefore help explain when and why the characteristics and demands of sport, which is typically a collective endeavour, even for athletes who compete in individual sports, pose a potential threat to mental health, as well as help identify potential opportunities for intervention.

In the sections that follow, we first provide a short introduction to the social identity approach, noting that detailed outlines are available elsewhere (e.g. [15, 16]). Then, drawing on specific hypotheses offered by the social identity approach and evidence from extant research, we outline three key points that speak to the value of using a social identity lens in the context of athlete mental health. In the process, we focus on (a) practical steps that relevant stakeholders (e.g. coaches, support staff, clubs and governing bodies) could take to support athlete mental health that follow from a social identity analysis and (b) key avenues to explore in future research to further test our propositions and advance understanding. In recognition that sport-specific stressors are more pronounced at higher levels of competition [17], our analysis is often oriented toward, and arguably most relevant for, elite athletes. Nevertheless, our points also apply to an analysis of mental health for athletes competing at sub-elite levels.

Consistent with contemporary perspectives, we conceptualise mental health as a continuum [18–20]. According to continuum models, the positive end of the mental health continuum is characterised by experiencing high levels of flourishing (i.e. high well-being and functioning) and (typically) an absence of a mental illness or disorder. The negative end of the continuum is characterised by languishing (i.e. poor well-being and functioning) and often the presence of a mental illness or symptoms thereof. Although the presence of well-being and the absence of a mental illness are separable constructs ([20]; see also [21, 22]), for the purposes of our analysis, we are interested in both maximising well-being *and* minimising illness. A critical implication of this contemporary perspective is that mental health is relevant for *everyone* (not just people with a mental illness). In the same way that people—particularly elite athletes—might aim for peak physical performance, so too can they aim for peak mental flourishing. Indeed, mental health and performance are often thought to go hand-in-hand for athletes [23]. This makes supporting athlete mental health an imperative, even for stakeholders whose primary role is to maximise athlete performance.

Our analysis is also consistent with ecological models that emphasise the ways in which health is shaped by several (interacting) layers of influence [24]. For example, recent applications of ecological models to sport have specified four layers of factors that influence elite athlete mental health: the athlete themselves (e.g. their coping skills), the microsystem (e.g. the athlete’s coaches and friends), the exosystem (the individual sport) and the macrosystem (the international sporting environment, public and social media [4, 25, 26]). We argue that the social identity approach provides a theoretical framework that can help explain *how* and *why* factors at these different layers affect athlete mental health.

## The Social Identity Approach

The social identity approach starts by recognising an individual’s capacity to define themselves both in terms of their personal identity (as ‘I’ and ‘me’) and their social identities (as members of the various groups to which they belong [13]). The impact of defining oneself as a group member was first observed in the ‘minimal group’ studies [27]. These studies assigned participants to groups based on arbitrary criteria, and researchers consistently found that participants favoured fellow members of their group (i.e. in-group members) over members of another group (i.e. out-group members) in their allocation of rewards (e.g. small sums of money). Indeed, participants tended to allocate rewards in ways that maximised the relative gain of the ingroup over the outgroup (rather than ways that maximised overall gain, overall ingroup gain or fairness). The key implication of these seminal findings was that, even in the most minimal or ‘stripped-back’ group conditions, defining oneself as a group member is sufficient to impact one’s thoughts and behaviours, such that people are guided by group needs and priorities rather than (only) individual ones. In other words, the minimal group studies indicated that people behave like group members not because of other phenomena such as interdependence, interpersonal emotional ties or an awareness of group norms but merely because they see themselves *as group members*.

When subsequently developing the social identity approach, Tajfel and Turner [13] sought to build on this key finding by providing a comprehensive analysis of the wide-ranging and meaningful ways through which defining oneself as a group member (e.g. as a member of one’s sports team), and subjectively internalising that group membership such that it becomes an important part of ‘who you are’ (i.e. developing a strong social identity as a member of a particular group or team) affects the way people think, feel and behave (see also [14]). These analyses are highly relevant to sport. Sport is a context in which groups are not only present, but often centre stage (see also [16, 28]). Even athletes competing in individual sports interact within a group microsystem, such as training groups and broader entourages of support (e.g. sports science and sports medicine). For team sport athletes, the success of one’s team is often seen as a marker of individual achievement [29], and a team’s culture and climate are known to have a key influence on individual athlete mental health [30, 31]. In the following sections, we introduce some of the specific propositions of the social identity approach, with a focus on their relevance and utility to understanding and promoting athlete mental health. Our analysis therefore seeks to build on recent attempts to highlight and synthesise the approach’s potential to advance understanding of key topics in sport (and exercise) psychology. In particular, here, we go beyond previous reviews which either explore social identity in sport or exercise but do not consider mental health [16, 28, 32] or provide an introduction to the topic for a generalist audience [33]. Importantly too, and as noted above, key and novel goals of the present article are to (a) provide practical recommendations for stakeholders seeking to support athletes’ mental health and (b) identify areas in which more research is needed and ways to fill these gaps. These are summarised in Table 1.

**Table 1:** Summary of recommendations for stakeholders seeking to support athlete mental health and key avenues for future research

Key point	Applied recommendations for stakeholders seeking to support athletes’ mental health	Recommendations for future research
1. Social identities can act as psychological resources that support athlete mental health	Use tools such as Social Identity Mapping to develop a comprehensive picture of athletes’ group memberships Monitor athletes’ social identification with their team, and social group connections outside their team, on a continuous basis to help identify athletes who may be at risk of poor mental health	Conduct longitudinal research examining how the quality and quantity of athletes’ group memberships affect their mental health over time Examine the mechanisms through which group memberships confer mental health benefits for athletes Examine the mental health effects of developing and strengthening athletes’ social group connections (e.g. through Groups 4 Health style interventions)
2. Social identities are critical to athlete mental health during and after sporting transitions	Increase athletes’ awareness that developing and maintaining multiple social group memberships is crucial for their mental health in the lead up to transitions Provide athletes opportunities, and build their capacity, to develop and maintain multiple group memberships. Treat this as an ongoing imperative, to ensure athletes have a wealth of social group capital to draw upon when they face expected or unexpected transitions Educate athletes that group memberships can be diverse and need not take up large amounts of their time	Test the predictions of the Social Identity Model of Identity Change in the context of a wide array of sporting transitions, and in relation to a wide range of mental health outcomes for athletes Test all Social Identity Model of Identity Change’s predictions in the context of single studies. That is, assess the role that social identity maintenance, gain and compatibility play in supporting athlete mental health across transitions Use nuanced measures of group memberships to assess how the number of group memberships athletes possess, and the importance they attach to them, interact to influence how successfully athletes navigate transitions
3. Social identity leadership can facilitate athlete mental health	Demonstrate identity leadership by using: 1. Evidence-based strategies (e.g. inclusive language, emphasising similarities between team members, highlighting the importance of the team in achieving success and framing achievements as collective successes) 2. Activities from identity leadership training programmes (e.g. work with athletes to identify team values and behaviours that align with those values, then demonstrate those behaviours)	Conduct longitudinal research assessing the relationship between sporting leaders’ engagement in identity leadership and indicators of athlete mental health and mental ill-health over time Examine how changes in leadership affect athletes’ mental health, with a focus on whether incoming leaders can buffer negative mental health impacts and support athletes to flourish after leadership changes by engaging in identity leadership Test whether (3R/5R style) identity leadership training programmes have meaningful and long-lasting benefits for athlete mental health, and the mechanisms through which these potential benefits arise

## A Social Identity Approach to Athlete Mental Health

### Key Point 1: Social Identities can act as Psychological Resources that Support Athlete Mental Health

In the last two decades, the importance of social connectedness for health has become increasingly well recognised. In two seminal meta-analyses, Holt-Lunstad and colleagues [34, 35] found evidence that poor-quality social relationships (conceptualised in terms of a lack of integration and support) have a comparable impact on mortality risk to well-established risk factors such as substance use and obesity. Similarly, there is strong evidence, including from large population studies and systematic reviews, that social isolation and loneliness are key risk factors for depression, while social relationships are protective (e.g. [36, 37]). Indeed, leading theories of suicide reference the critical components of belonging and burdensomeness [38, 39], which are both relational in nature.

Social identity researchers have proposed that the health benefits people gain from social connection are often attributable to belonging to social *groups* that are meaningful to them (i.e. from possessing social identities), and (thus) that it is social identities that hold the key to unlocking a ‘social cure’ [40]. More specifically, they have argued that social identities provide people with psychological resources on which they can draw that help protect and enhance their mental (and physical) health [41, 42]. In line with this, there is evidence that these resources include a sense of belonging, meaning, esteem and purpose—fundamental psychological needs that, when satisfied, support mental health (e.g. see [43–45]). In particular, across three studies, Greenaway et al. [46] found consistent evidence that satisfaction of these needs mediated the relationship between social identities and depression. For example, in an experiment, participants who were asked to recall and write about an instance where they had gained a social identity reported greater need satisfaction and fewer depression symptoms than participants who were asked to recall and write about an instance where they had lost a social identity. Sensitivity analyses across all studies further indicated that the benefits of social identities were not reducible to the satisfaction of any one need. Rather, they were underpinned by need satisfaction in a global sense.

Researchers have also found evidence that another resource that social identities can provide is social support. In short, research suggests that defining oneself as a group member, and internalising that group membership such that it becomes an important part of who you are, provides a psychological basis for social support: people are both more likely to feel supported by fellow group members if they strongly identify with the group [47–50], and indeed group members are more likely to actually offer another person support if they perceive them as a fellow ingroup member ([51]; see also [52]).

Notably in the present context, recent research has also provided initial evidence that social identities provide resources that support mental health among athletes. In a year-long longitudinal study, Graupensperger et al. [53] assessed college athletes’ (*N* = 697) social identification as a member of their sports team and well-being (using a measure that comprised indices of life satisfaction, happiness and subjective health) at three timepoints. Multi-level analyses showed that participants’ well-being at the final timepoint was greater among those who (a) strongly identified as a member of their sports team at baseline and (b) experienced an increase in the strength of their social identification during the year.

Graupensperger et al.’s [53] findings point specifically to the benefits of fostering athletes’ social identification as a member of their team—a finding supported by a recent meta-analysis, which found that social identification building interventions (in diverse contexts) show medium-large effect sizes on various mental health and ill-health indicators (e.g. Hedges’ *g*s were 0.66 for well-being, 0.58 for reduced depression and 0.49 for reduced stress [54]). However, social identity theorising and research suggest that an athlete’s social identity as a member of their sports team is not the only social identity that those seeking to support their mental health should attend to. Consistent with the notion that social identities provide mental health-enhancing resources, researchers have further proposed a ‘more the merrier’ effect, such that (providing the identities are positive and compatible) possessing *multiple* social identities should amplify the benefits people experience because it means they have a greater number and range of resources on which they can draw (e.g. more groups to help satisfy their psychological needs, and more sources of social support [41, 42]). This proposition has been widely supported for diverse indicators of mental health. For example, research in non-athlete samples has found that belonging to multiple social groups can enhance self-esteem [55], life satisfaction [56] and quality of life [57], and buffer against, and help alleviate the symptoms of, depression [58, 59]. Indeed, speaking to the magnitude of these effects, using data from a large longitudinal cohort study, Cruwys et al. [58] found that depressed participants who joined one group reduced their risk of depression relapse 4 years later by 24%, while those who joined three groups reduced their risk of relapse by 63%.

A further powerful demonstration of the benefits of belonging to multiple social groups has been provided by experimental studies that have found that merely making people’s group memberships salient can provide them with a psychological ‘boost’ [60–62]. This includes research focusing on sporting tasks, where researchers have found that making participants’ group memberships salient can increase their capacity to ‘bounce back’ from the types of setbacks that athletes commonly face (e.g. performance setbacks and task failure). For example, in two experiments, Green et al. [60] divided participants into three conditions: two active conditions, where participants were asked to reflect on, and describe the importance of, either one or five of their group memberships, and a control condition, where participants did not complete a group membership manipulation. All participants then completed a golf-putting task before being told that their performance placed them in the bottom 30% of participants in the experiment so far (i.e. a performance setback). Participants were then given a free period, in which they could practice the task, rest or engage in other activities. After this, they attempted the golf task a second time. The results showed that participants in the group memberships conditions demonstrated greater resilience following the setback than participants in the control condition. That is, they completed a greater number of practice attempts during the free period (Studies 1 and 2) and demonstrated greater performance improvements during the second golf task (Study 2).

#### Key Point 1: Applied Implications and Directions for Future Research

Taken together, the findings summarised above provide promising initial evidence that social identities may represent a powerful resource for athletes. From an applied perspective, this prior work suggests that efforts to develop a complete understanding of an athlete’s mental health might be enhanced by gaining a picture of, and monitoring, their group memberships. In the same way that coaches and sports science support staff routinely monitor athletes’ physical loads to identify those who are at higher risk of physical injury [63], they may wish to monitor athletes’ social identification with their team, and social group connections outside the team, to identify those who may be at risk of poor mental health. Indeed, an additional benefit of this approach is that, compared with direct measures of mental health, athletes may be more willing to complete group memberships measures honestly (given the lesser likelihood of associated stigma [11, 12]).

One tool that could be used for the purpose of measuring and monitoring athletes’ social identities is Social Identity Mapping (SIM [64]). Social Identity Mapping supports people to create a visual representation or ‘map’ of their social group memberships. People often find that completing SIM provides self-insight and can even be a semi-therapeutic exercise in itself [65]. However, SIM can also be used to calculate numerical indicators of the quantity and quality of a person’s social group memberships (on dimensions including subjective importance, positivity, similarity and compatibility), and changes over time in these indicators (particularly on ‘quality’ dimensions) have been found to correspond to changes in mental health [64, 66]. Research has demonstrated the convergent and discriminant validity of the tool and found that people find both the paper and pencil and online versions easy to use [64, 66]. Cascagnette et al. [67] also provided initial evidence for SIM’s usefulness to help understand sports team dynamics. However, while these researchers’ focus was on SIM’s utility as a tool to enhance team performance, we argue that its potential application in sport extends beyond this and includes enabling insights into factors that also have important implications for athletes’ mental health (i.e. the quality and quantity of their social group connections).

Notwithstanding these applied opportunities, further research is needed to build a more comprehensive understanding of the mental health benefits that social identities can provide athletes, and to address unanswered questions, including exactly how social identities can provide mental health benefits to athletes. There would thus be particular value in (a) further longitudinal research examining relationships between the quality and quantity of athletes’ (particularly elite athletes’) group memberships and their mental health and (b) research that focuses on identifying the mechanisms through which group memberships confer mental health benefits for (elite) athletes. That is, research is required to ascertain whether the ‘resources’ that have been shown to underpin the mental health benefits of group memberships in non-sporting samples (e.g. increased need satisfaction and perceived social support [46, 47, 52]) similarly underlie the mental health benefits that have begun to be observed in athlete samples [53].

Research examining the mental health effects of developing and strengthening athletes’ social group connections is also needed. Outside sport, research has provided consistent evidence for the mental health benefits of a programme with these goals. Run in small groups, Groups 4 Health is a manualised five-module programme that focuses on building group-based belonging. Across multiple sessions, participants are guided through activities that aim to help them understand the value of groups for health, map out their group memberships, get the most out of existing group memberships and effectively develop new group memberships. In various samples (e.g. community members with mental illness, students beginning university and young people with depression), Groups 4 Health has demonstrated wide-ranging mental health benefits for participants, including improved well-being, reduced social anxiety and comparable reductions in depression symptoms to cognitive behavioural therapy (the gold-standard treatment [68–70]). Examining the benefits of Groups 4 Health for athletes would provide a strong causal test of the social identity approach’s utility as a guiding theoretical framework for efforts to improve athletes’ mental health. In Table 2, we provide a breakdown of the five Groups 4 Health modules and note specific considerations for, and potential benefits of, each module for athletes.

**Table 2:** Overview of Groups 4 Health, and of specific considerations for, and potential benefits of, the programme for elite athletes

Module	Summary of goals/content	Specific considerations and potential benefits for elite athlete samples
Appreciating groups	Raising awareness of the value of groups for health and of ways to harness this	It may be necessary and valuable to debunk the perceptions that (a) eschewing all social groups in favour of an exclusive focus on one’s sport is beneficial and (b) (non-sporting) groups unavoidably take up too much of an athlete’s time
Mapping groups	Developing social maps to identify existing connections and areas for social growth	This activity may (a) help staff identify athletes who are particularly socially disconnected and most in need of support and (b) help athletes themselves realise if they have become socially disconnected (e.g. from their non-sporting groups) and prompt them to act
Strengthening groups	Training skills to maintain and utilise existing networks and reconnect with valued groups	This phase could fruitfully include both (a) a focus on developing athletes’ skills to connect with their existing broader social networks (including non-sporting groups) and (b) activities designed to strengthen connections between athletes who complete the programme together (e.g. teams could complete the programme together and activities could explicitly focus on strengthening team dynamics)
Extending groups	Using the programme group as a platform for new social connections and to train effective engagement	Athletes completing the programme together may identify shared (non-sporting) interests. Here, they could be encouraged to pursue these interests together (e.g. join a new group that supports that interest). Athletes could also work with each other, and programme facilitators, to troubleshoot how to fit other interests around their sporting commitments
Sustaining groups	Reinforcing key messages and troubleshooting	‘Booster’ sessions that revisit and seek to maintain and strengthen connections among team members may be particularly valuable following periods where several team personnel changes have occurred (e.g. after transfer windows or trade periods)

Along these lines, we also note recent evidence for the benefits of sport-specific mental health interventions for athletes that have involved athletes’ ‘supportive others’ (e.g. family members, coaches, teammates [71, 72]). In particular, researchers have found that having more supportive others involved throughout such interventions as support agents (e.g. who provide athletes with encouragement and help with problem solving) is associated with greater reductions in athletes’ psychiatric symptoms [71]. By its nature, Groups 4 Health is conducted in groups. It therefore provides an opportunity to involve supportive others not just as support agents, but as active participants in the programme. Indeed, as noted in Table 2, sports teams could complete the programme together and teammates could be encouraged to work together to achieve programme goals (e.g. by pairing up and joining new social groups together). It is therefore possible that, by directly harnessing the power of supportive others in this way, the benefits of Groups 4 Health could be even greater for athletes than those that have been observed for general population samples (where programme participants do not typically know each other [69, 70]).

### Key Point 2: Social Identities are Critical to Athlete Mental Health During and After Sporting Transitions

One reason that efforts to monitor and develop group memberships may be particularly valuable for athletes is because this population is known to be at high risk of belonging to few social groups [73–75]. Along these lines, several studies have highlighted the tendency for athletes (at all levels, but elite athletes in particular) to define themselves strongly—and in some cases exclusively—on the basis of their athletic role (i.e. possess a strong athletic identity [75–77]). They often justify this on the basis of needing to ‘fully commit’ and ‘make sacrifices’ to achieve their goals [78, 79]. However, possessing a strong athletic identity is often accompanied by a ‘closing off’ of other identities [73, 75], and has been associated with an increased risk of athletes experiencing burnout [80, 81] and being more vulnerable to mental ill-health conditions such as depression when their athletic identity is threatened—for example, when they are temporarily or permanently unable to participate in sport because of injury, illness, de-selection or retirement [82, 83].

Building on the previous section, these findings further speak to the dangers of athletes failing to maintain multiple social identities and indicate that one time when social identities are particularly important for athletes’ mental health (and when a lack of them can pose a particular threat to athletes’ mental health) is during and after *transitions*. Broadly conceptualised as turning phases in athletes’ careers that involve appraising, and coping with, specific demands leading to successful or less successful outcomes [84, 85], sporting transitions include sustaining serious injuries [86, 87] and retiring from sport [83, 88], as well as transferring between clubs [89], progressing from youth to senior [90, 91] and amateur to professional [92] sport, and relocating to a residential high-performance centre [93, 94]. There is evidence that these transitions, including those that are typically considered positive, are often associated with increased stress and reduced mental health (e.g. [95, 96]). As such, sporting transitions research has sought to understand the factors that facilitate more positive transition experiences. Most of this research has focused on the role of individual-level factors (e.g. the athlete’s level of education), demographic variables (e.g. the athlete’s age) and the characteristics of the transition itself (e.g. whether it is voluntary [88, 97, 98]). However, the social identity approach offers an alternative perspective on why transitions pose a particular threat to people’s mental health, which is articulated in the Social Identity Model of Identity Change (SIMIC [99]).

**Fig. 1:**
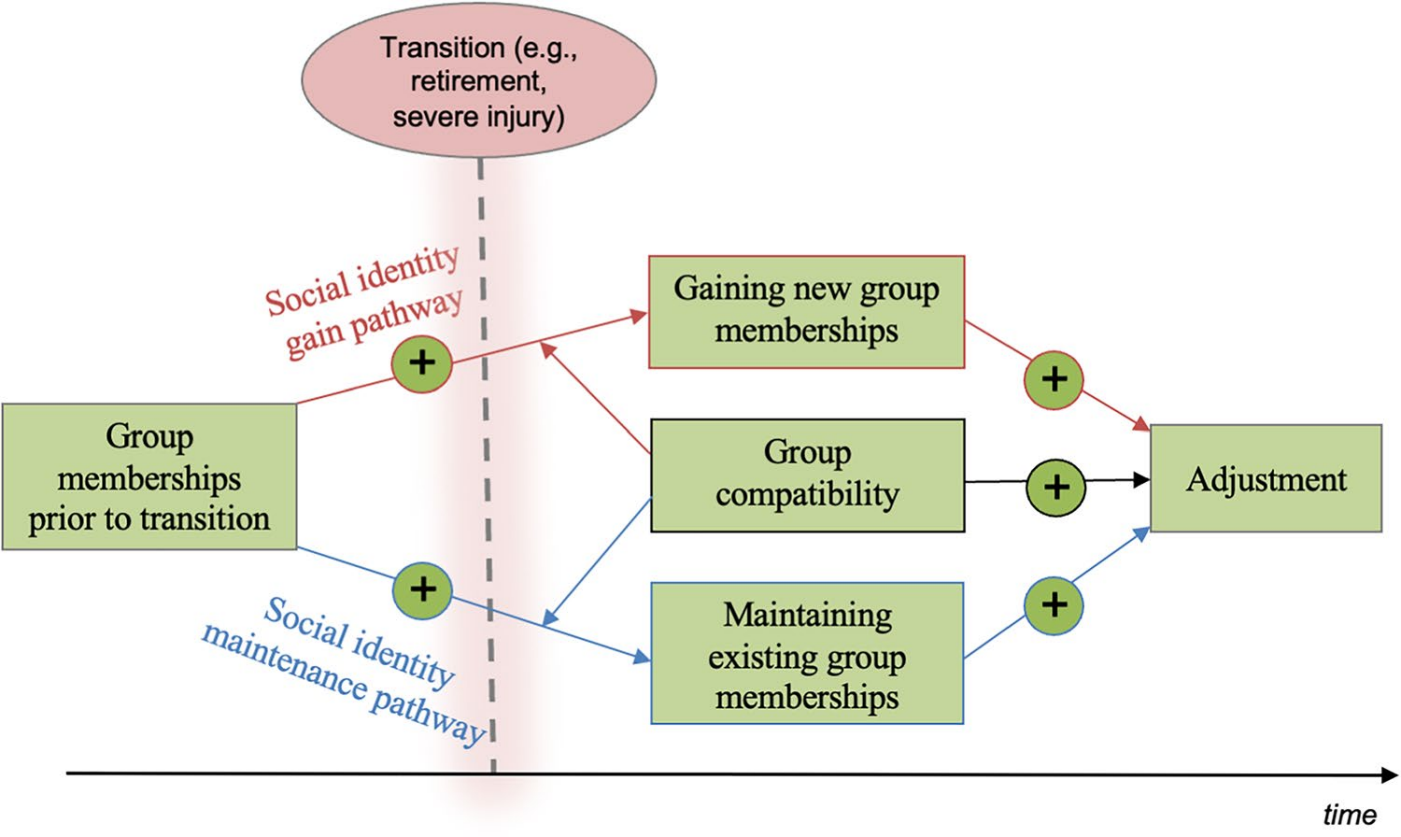
Schematic overview of the Social Identity Model of Identity Change (adapted from Haslam et al. [99], with permission)

The central tenet of SIMIC is that transitions typically involve a process of *social identity change* and that much of the (typically negative) impact that transitions have on health is due to these identity-based changes. In particular, while transitions can lay a foundation for people to connect to new groups, they also often result in people’s connections to their existing groups being lost or weakened. The potential for such social identity changes to occur is apparent for each of the sporting transitions mentioned above. For example, transferring clubs or relocating to a residential high-performance centre can facilitate new group memberships for an athlete (e.g. as a member of their new team), but at the same time require geographical relocation that may disconnect them from both sporting and non-sporting groups. According to SIMIC, both *maintaining* existing group memberships and *gaining* new memberships during transitions are pathways that are critically protective for a person’s health. Importantly too, SIMIC proposes that having more group memberships before the transition occurs increases the likelihood that a person will both (a) maintain at least some of those group memberships and (b) gain more group memberships (because they are more likely to have the attitudes and social skills that make them willing and able to join new groups). Finally, SIMIC also proposes that successful adjustment to a transition is further supported if the group memberships that a person maintains and gains are *compatible* (e.g. in terms of their norms and values). For example, an athlete transitioning from amateur to professional sport might find that their new teammates adhere to a disciplined lifestyle typical of a professional athlete. Those norms might, however, be incompatible with norms among groups of their former amateur teammates, who regularly organise late-night social events (for a schematic overview of SIMIC, see Fig. 1).

The propositions of SIMIC have been supported in the context of various transitions. For example, researchers have found evidence that maintained and new group memberships support people’s mental (and, at times, physical) health following retirement [56, 57, 100], beginning university [101], becoming a mother [102] and experiencing a brain injury [103]. Most notably in the present context, two recent studies have provided initial support for some of the model’s key predictions among athletes navigating sporting transitions.

First, using a longitudinal design, Rees et al. [104] examined junior elite cricketers’ (*N* = 257) experiences of transitioning into high-performance pathways. They found that those who (a) possessed a greater number of pre-transition group memberships and (b) gained a greater number of new group memberships post-transition had more positive outcomes following transition than those who belonged to, or gained, relatively fewer group memberships. Specifically, both pre-transition and new group memberships positively predicted the cricketers’ post-transition performance (as rated by their coaches). Moreover, speaking to the implications for mental health, both pre-transition and new group memberships also predicted participants’ greater post-transition life satisfaction and positive affect, and new group memberships positively predicted participants’ self-esteem.

Along similar lines, in a cross-sectional study of retired elite athletes from Western (*N* = 215) and Eastern (*N* = 183) geographical regions, Haslam et al. [105] found that participants who maintained or gained a greater number of group memberships post-retirement tended to adjust to retirement better, as indicated by higher scores on an ‘adjustment index’ that comprised measures of participants’ life satisfaction, depression and physical health. There was also some evidence from mediation analyses that greater meaning in life and perceived control were the resources that underpinned these benefits.

#### Key Point 2: Applied Implications and Directions for Future Research

From an applied perspective, the findings reviewed in Sect. 3.2 further underscore the need to increase athletes’ (a) awareness that developing and maintaining multiple group memberships is crucial for their mental health and (b) opportunities and capacity to do this, particularly in the lead up to transitions. Indeed, given that many sporting transitions (e.g. sustaining serious injuries and transferring clubs) can be unexpected, athletes and those responsible for supporting their mental health (e.g. coaches, support staff, and athlete well-being and engagement providers) must be proactive and treat maintaining social group connections as an ongoing imperative. In line with SIMIC’s predictions, this will ensure that when athletes do face a transition, they have a wealth of social group capital to draw on to help them navigate it effectively [99].

It is also important to note that, while athletes may be hesitant about the time commitment associated with belonging to multiple (especially non-sporting) social groups, these groups need not take up large amounts of their time and this should be emphasised to athletes and those who support them. Group memberships do not only include recreational activity groups; they can also include friendship groups, special interest groups, opinion-based groups and demographic groups. Moreover, from a social identity perspective, the value that a person attaches to a group membership (i.e. the strength of their social identification as a group member) is a stronger determinant of that group’s benefit to them than the amount of social contact they have with other group members or how frequently they engage in group activities [99, 106]. Thus, it is possible even for athletes whose lifestyle unavoidably detaches them physically from some of their social groups (e.g. professional tennis players who are travelling on tour for 10 + months a year) to possess and maintain valued social identities that can support their mental health. Indeed, maintaining social connections is now arguably easier than it ever has been thanks to technology, widespread Internet access and social media platforms (e.g. tennis players on tour can continue to interact with groups of their friends at home via WhatsApp groups).

There are several potential avenues for future research that would facilitate a more complete understanding of the value of a social identity-based analysis of sporting transitions and shed greater light on how to utilise this analysis to better support athletes’ mental health during these periods. First, research is needed that tests SIMIC’s predictions (a) in the context of a wider array of sporting transitions and (b) in relation to a wider range of mental health outcomes for athletes. For example, moving beyond measures of general well-being that have been the predominant focus to date, research could focus on outcomes such as psychological distress, sleep disturbance or anxiety. Moreover, extant research (both within and outside sport) has tended to test only some of SIMIC’s propositions. In particular, the compatibility hypothesis in the model has rarely been examined in empirical research. Studies that assess the complete model are needed to gain a comprehensive understanding of its value. Indeed, such studies are particularly important given growing evidence from outside sport that SIMIC’s pathways diverge in their relative importance across different types of transitions (e.g. evidence suggests that gaining new group memberships is a particularly strong predictor of a positive retirement transition in non-sporting samples [56]).

Relatedly, there is also a need for research that uses more nuanced measures of group memberships. Although SIMIC primarily focuses on how *many* social group connections people have—and researchers have thus also tended to focus on this in empirical research—as alluded to above, the quality of those group memberships (i.e. how strongly people identify with them) is critical in theorising about why groups have benefits [99, 106]. Researchers may therefore seek to examine the ‘trade-off’ between belonging to a greater number of less valued groups versus a smaller number of highly valued groups (utilising measures that capture both the quantity and quality of people’s group memberships; e.g. see [107]). This would help guide efforts to more accurately identify athletes who may be at potential risk of adjustment problems in the context of transitions, as well as poorer mental health in general, and who may therefore be the most important to offer support to or recruit for interventions.

### Key Point 3: Leadership Informed by a Social Identity Approach Can Facilitate Athlete Mental Health

Beyond the sporting context, it is well established that negative work group environments pose a major threat to people’s mental health [108, 109]. Although numerous factors can contribute to groups becoming ‘toxic’ [110], the individuals who often have the greatest capacity to influence a group’s culture and climate — either positively or negatively — are *leaders*. Recognising this, researchers have devoted substantial attention to understanding how, by engaging in different leadership styles and behaviours, leaders can (a) foster an environment that supports group members to flourish (e.g. [111, 112]) and (b) help protect against (or, conversely, contribute to) work groups becoming toxic (see [113] for a review). Very few studies with these goals have been conducted in sport (see [114] for an exception). However, researchers have highlighted the important influence that team environments can have on athlete mental health [30], and suggested that coaches have a key role to play in fostering a team environment that supports athlete mental health [5, 115]. Instances of elite athletes highlighting the negative cultures they have perceived in high-performance teams and programmes have also become more frequent (e.g. [116, 117]), with failings in leadership often cited as a reason for such cultures developing (including in independent investigations [118]).

The social identity approach provides an analysis of effective leadership that appears particularly well suited to explaining how leaders can create a positive group environment. This is not least because, contrary to many leadership theories, which focus on the leader in isolation and emphasise traits or behaviours that they should possess or demonstrate *as individuals* (e.g. see [119–121]), the social identity approach to leadership places the group at the heart of its analysis. More specifically, it proposes that leaders’ capacity to facilitate positive group member outcomes and mobilise group members toward common goals rests on the extent to which they are able to foster a sense of shared identity (i.e. a shared sense of ‘us’) among group members [122]. To this end, it argues that leaders should strive to engage in actions and activities that: (1) demonstrate that they *represent* the group’s identity (i.e. what it means to be one of ‘us’); (2) *advance* the group’s identity and interests; (3) help *create* a sense of shared group identity and define what it means to be a group member; and (4) help *embed* the group’s identity in reality (e.g. by providing practical activities that enable members to ‘live out’ their shared identity; see also [123]).

Numerous studies have provided evidence for wide-ranging benefits of leaders adhering to these four principles. For example, researchers have found that identity leadership can help mobilise political support [124, 125], increase organisational performance [126] and lay a foundation for group therapy patients to experience better therapeutic outcomes [127, 128]. Most relevant in the present context, researchers have also found evidence that identity leadership can help buffer against poor mental health. For example, in a two-wave study, Steffens et al. [129] found that manual workers’ (*N* = 338) perceptions of the extent to which their workgroup leader created a strong sense of shared social identity in their work team (i.e. acted as an ‘identity entrepreneur’) predicted the workers’ reduced burnout 10 months later. The researchers also found that (a) participants’ identity entrepreneurship ratings predicted their reduced subsequent intentions to leave the organisation (a common response to working in a negative environment [130]), and importantly, (b) no evidence for the reverse pathways.

The social identity leadership approach is also gaining traction as a popular framework in sport. Here, studies have found evidence that athletes experience various benefits to the extent that their coach, captain and informal team leaders engage in identity leadership and thus help strengthen athletes’ social identification as a team member. For example, athletes tend to participate in team training sessions more frequently [131, 132], demonstrate greater commitment to team goals [133] and report performing better ([134]; see [135] for a recent review). There is also some promising evidence that identity leadership can facilitate positive mental health outcomes for athletes.

First, in a cross-sectional survey study of national and regional level handball players (*N* = 289), Fransen et al. [136] found support for a path model in which players who perceived that their coaches, captains and informal athlete leaders engaged in more identity leadership reported greater social identification as a team member and greater perceived psychological safety and, through these serial mediators, lower burnout and greater self-rated global health (on a measure that comprised indicators of their physical health, state of mind and energy levels). Notwithstanding the limitations of the study’s cross-sectional design, this initial evidence that leaders can create a psychologically safe environment (i.e. characterised by interpersonal trust, mutual respect and acceptance, and in which individuals feel comfortable taking risks [137]) by engaging in identity leadership is particularly promising. This is because other recent research has found that elite athletes report fewer mental ill-health symptoms, lower psychological distress, higher well-being and a reduced risk of eating disorders to the extent that they perceive their sporting environment is psychologically safe [26, 31].

Second, speaking to the causal effects of identity leadership, researchers have found initial evidence that identity leadership training programmes can have downstream benefits for athlete mental health. The structure of these programmes, which are often referred to as ‘3R’ or ‘5R’ programmes, has varied slightly across studies. However, at their heart are three sets of activities (grouped under the headings ‘Reflecting’, ‘Representing’ and ‘Realising’) that leaders are encouraged to conduct with their team that aim to develop and demonstrate their identity leadership (see [138] for a detailed overview). The first set of activities (in the ‘Reflecting’ phase) typically aims to support the leader to understand their team’s identity and dynamics (e.g. through asking each player to draw a map of their team identity). The next set of ‘Representing’ activities then aims to enable leaders to represent and champion the team identity (e.g. they might work with their team to identify team values and behaviours that align with those values). Following this, in the ‘Realising’ phase, leaders will be in a position, and are encouraged, to adapt their behaviours and the environment in ways that support the identity, values and goals of the team to be realised (e.g. by demonstrating value-consistent behaviours and reinforcing team values during team talks or through posters placed in the changing room). In a recent randomised controlled trial, Mertens et al. [139] found that delivering an identity leadership programme to athlete leaders in ‘semi-elite’ basketball teams increased the players’ self-rated global health (on the same scale used by Fransen et al. [136]) and reduced their burnout (in addition to facilitating positive performance-related outcomes; see also [133]).

#### Key Point 3: Applied Implications and Directions for Future Research

The key role of sporting leaders in supporting versus undermining the mental health of their athletes is becoming increasingly well recognised, with much of this impact appearing to occur via the crucial role these leaders play in shaping the team environment [5, 115]. The findings reviewed above suggest that the social identity leadership approach can help explain when and why leaders’ impact on the team environment, and thus on athletes’ mental health, is positive or negative. Specifically, they suggest that athletes’ mental health is likely to benefit to the extent that leaders foster a shared sense of identity (or ‘us-ness’) within the group they are leading. In line with calls to develop a greater understanding of the specific ways through which leaders can achieve this [135, 140], researchers (in a range of contexts) have proposed and tested various strategies.

First, researchers have identified that using inclusive language (i.e. ‘we’ and ‘us’ rather than ‘I’ and ‘me’) when communicating with group members can help leaders present themselves as ‘one of us’, cultivate a sense of shared identity and facilitate positive outcomes [124, 126, 141]. For instance, retrospective analyses of leaders’ communications have found that organisational leaders’ use of inclusive language positively predicts organisational performance [126] and that political leaders’ use of inclusive language positively predicts candidates’ election success [124]. There is also evidence that people tend to identify more strongly with social groups in which they perceive group members are similar to each other on dimensions that are relevant in the given context (e.g. in terms of their physical fitness in an exercise class [142, 143]). It therefore follows that emphasising team members’ similarities on such dimensions (e.g. in terms of their attitudes toward training or their sporting goals), as well as ways in which the team as a whole is positively distinct from other teams [13, 144], may be advantageous for leaders seeking to build social identification. Finally, researchers have also found evidence for the benefits of leaders expressing confidence in the team, highlighting the importance of the team in achieving success and framing achievements as collective successes ([141, 145]; see also [146]). This includes experimental studies using sporting tasks where such messages have been shown to facilitate improvements in team members’ effort during a cycling time trial [141] and performance during a skill-based soccer task [145]. Leaders may wish to communicate such messages internally (e.g. during team talks and training sessions) as well as externally during interactions with the media (e.g. in interviews and press conferences), where their narratives can have a meaningful impact on athlete outcomes [144].

Identity leadership training programmes also provide some examples of how sporting leaders (e.g. coaches and captains) can engage in identity leadership and build social identification. That is, like the leaders recruited for the programmes discussed above [133, 139], these leaders could utilise activities designed to help them understand, represent and champion their team’s identity. For example, they could run workshops with their players in which they aim to identify team values and behaviours that align with those values. The leaders could then show they are representative team members by demonstrating the agreed-upon behaviours. Leaders could also look to these programmes for further examples of concrete behaviours that might be fruitful. For example, Fransen et al. [147] recommended that leaders in their programme encourage team members to create a team WhatsApp group to help facilitate interactions and communication among members outside team sessions and help strengthen their social identification with the team (for other iterations of identity leadership programmes, see [148, 149]).

Despite the promise of the identity leadership framework, it is important to note that empirical research examining the link between identity leadership and athletes’ (particularly elite athletes’) mental health remains in a nascent state and that much more research is therefore needed. Longitudinal research (e.g. over the course of a season or multiple seasons) assessing a wider array of mental health and ill-health indicators (e.g. life satisfaction, depression and psychological distress) would be particularly valuable. This would enable insights into the extent to which identity leadership can help with the tasks of both protecting athletes from mental ill-health *and* supporting them to flourish. Longitudinal designs would also be well suited to examining how changes in leadership (e.g. coaches being replaced, a common occurrence in elite sport) affect athletes’ mental health. In particular, research could test whether it is the leadership approach that incoming (vs outgoing) leaders adopt (e.g. aligned with the identity leadership or not) that underpins whether a leadership change positively or negatively affects athletes’ mental health. Research is also needed that builds on the promising initial evidence from Mertens et al.’s [139] study and tests whether the downstream effects of identity leadership training programmes include a meaningful, and crucially long-lasting, boost to a wider array of indicators of athletes’ mental health and ill-health, as well as the mechanisms through which these effects occur.

## Conclusions

The characteristics and demands of sport can pose a major threat to athlete mental health, particularly at the elite level. The social identity approach is a potentially fruitful framework through which to understand how and why these characteristics and demands can threaten athlete mental health. It has the potential to guide novel approaches that prevent mental ill-health among athletes, reduce their mental ill-health symptoms and support them to flourish. Through three key points, we have sought to illustrate the social identity approach’s utility in the context of athlete mental health. Our hope is that this provides those seeking to support athlete mental health with a new perspective and a different set of ideas from which to draw, and acts as a stimulus for researchers to expand the evidence base and develop a more comprehensive understanding of the social identity approach’s value in this context.
